# Design and Evaluation of Smart Textile Actuator with Chain Structure

**DOI:** 10.3390/ma16165517

**Published:** 2023-08-08

**Authors:** Ju-Hee Lee, Min-Woo Han

**Affiliations:** 1 Department of Mechanical Engineering, Dongguk University, 30 Pildong-ro 1 gil, Jung-gu, Seoul 04620, Republic of Korea; 2 Department of Mechanical, Robotics and Energy Engineering, Dongguk University, 30 Pildong-ro 1 gil, Jung-gu, Seoul 04620, Republic of Korea

**Keywords:** textile actuators, smart materials, chain structure, soft robotics, shape memory alloy (SMA)

## Abstract

Textiles composed of fibers can have their mechanical properties adjusted by changing the arrangement of the fibers, such as strength and flexibility. Particularly, in the case of smart textiles incorporating active materials, various deformations could be created based on fiber patterns that determine the directivity of active materials. In this study, we design a smart fiber-based textile actuator with a chain structure and evaluate its actuation characteristics. Smart fiber composed of shape memory alloy (SMA) generates deformation when the electric current is applied, causing the phase transformation of SMA. We fabricated the smart chain column and evaluated its actuating mechanism based on the size of the chain and the number of rows. In addition, a crochet textile actuator was designed using interlooping smart chains and developed into a soft gripper that can grab objects. With experimental verifications, this study provides an investigation of the relationship between the chain actuator’s deformation, actuating force, actuator temperature, and strain. The results of this study are expected to be relevant to textile applications, wearable devices, and other technical fields that require coordination with the human body. Additionally, it is expected that it can be utilized to configure a system capable of flexible operation by combining rigid elements such as batteries and sensors with textiles.

## 1. Introduction

An actuator is a fundamental component that defines the functionality and efficiency of a mechanical system as a driving unit that generates motion in response to an external signal. As technology advances, the significance of actuators that can perform a variety of roles increases, as does the need for and interest in biomimetic soft actuators.

Soft actuators are based on soft materials such as polymers, rubbers, and silicones that enable smooth and flexible movement and are thus up-and-coming technologies with a wide range of applications, including wearable and adaptive robots [1,2,3].

Particularly, an intelligent material that responds to changes in the external environment has the advantage of being easily combinable with the structure of a soft actuator [4,5,6,7]. Soft actuators employing a variety of intelligent materials are actively being developed because these intelligent materials impart actuating performance while minimizing the interference of flexibility. Considering it is composed of exclusively soft materials, it can be utilized in systems that involve high levels of freedom, safety, and the human body [8,9,10].

Developing fiber-type artificial muscles is one of the promising technologies for creating soft actuators. Imitating the tendon-pulling motion of the human body, artificial muscle fibers are designed to achieve a higher power-to-weight ratio than that of human tendons [11,12]. In the case of developing coiling-type artificial muscles made by twisting polymer fibers used as fishing lines, it was possible to lift objects 100 times heavier than human muscles [13].

Various studies are being conducted to develop a fabric structure made of continuous fiber patterns to diversify the actuation modes of these fiber-type artificial muscles and achieve high strain and actuating force. Several investigations have been conducted concerning the force generated per unit mass, displacement, and actuating mode based on the geometry of the fiber (e.g., the fiber’s twist condition), arrangement, and bundle configuration [14,15,16]. Particularly, fiber-based actuators can be easily developed in the form of textiles for clothing and are categorized according to weaving, knitting, and other techniques based on the composition and orientation of their fibers.

The weaving method, the most common textile manufacturing technique, is a process of crossing two or more types of fibers made of warp and weft. This method is also employed as a textile arrangement in the manufacture of fiber-reinforced composites, and these woven structures show the characteristics of robustness and stability. As the warp or weft fiber, engineering fibers such as glass fiber and carbon fiber could be utilized, as well as shape memory alloy (SMA) wire, a type of intelligent material [17,18,19]. By combining SMA wires with woven structures or directly manufacturing them into woven fabrics, applications for 3D-morphing soft actuators have been developed [20].

In order to implement a more complex mode of deformation, a knitting-type textile actuator capable of incorporating several spaces into the textile structure has also been designed. Knitting is a technique of forming fibers into a number of loops, and depending on the arrangement of loops, it is possible to create stress in different directions [21,22,23,24]. SMA wires were knitted into patches with various loop configurations, and their behavioral properties were explored, such as deformed shape and blocking force. By utilizing the deformation characteristics of each looping pattern, it is possible to mimic the blooming shape of various types of flowers [25], and it has also been demonstrated that it could be used as a soft gripper [26].

This research aims to develop a chain-based intelligent textile actuator capable of diverse modes of deformation. The intelligent fiber used in this study, thermal reactive SMA wire, has a linear uniaxial contraction–tension behavior as its fundamental mechanism. Based on the linear motion of the SMA, we could derive 3D deformation from the textile actuator when the fibers are repeatedly arranged in a specific pattern. Intelligent fibers in these structures generate multidimensional deformation when an electric current is applied to them due to the Joule heating supply.

To investigate the behavioral characteristics of the chain actuator in relation to the change in design elements, the size of the chain composing the actuator was varied, and the displacement was observed with different size of chains. In addition, experiments were carried out to determine the contraction and torsional deformation in relation to the amount of electrical current supply that produces the phase change in the SMA. Particularly, in order to examine the magnitude of the joule heating effects, the deformation that occurs in response to the current supply was observed in chronological order. Additionally, the chain actuator is capable of adding rows, and the increase in actuating force due to the addition of rows was investigated.

Finally, a crochet actuator combining a chain and a loop was proposed in order to make a chain actuator into a patch type. Similar to the chain actuator, the crochet actuator exhibited torsional behavior, and a new type of soft gripper was proposed by constructing the crochet actuator with four patches.

## 2. Chain Actuators

### 2.1. Design

**Figure 1:**
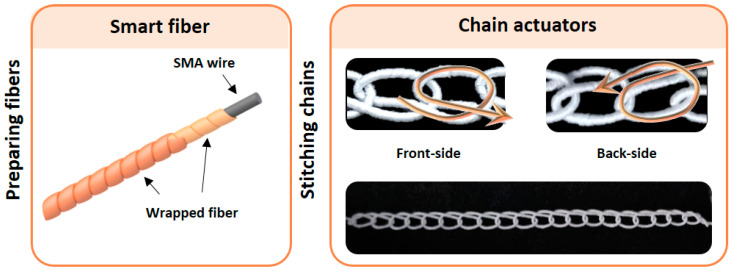
Fabrication of chain actuators.

The intelligent fiber of the chain-based textile actuator is made out of an SMA core (55 wt% Ni and 45 wt% Ti) wrapped with general fiber. When intelligent fibers are patterned into a textile, they can perform a diverse range of movements. Figure 1 depicts a detailed structural diagram of the smart fiber and chain structures.

### 2.2. Materials

**Figure 2:**
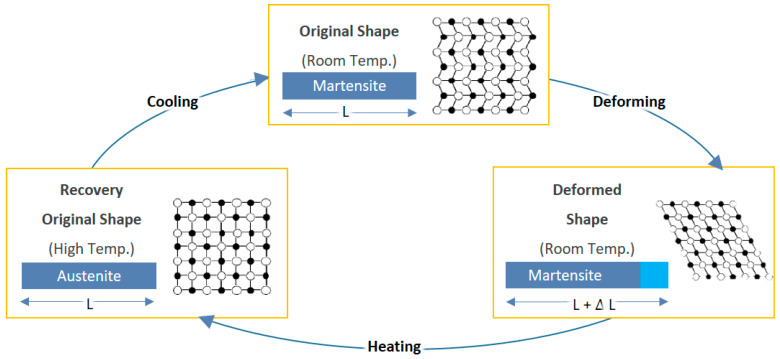
Phase transformation of SMA.

**Figure 3:**
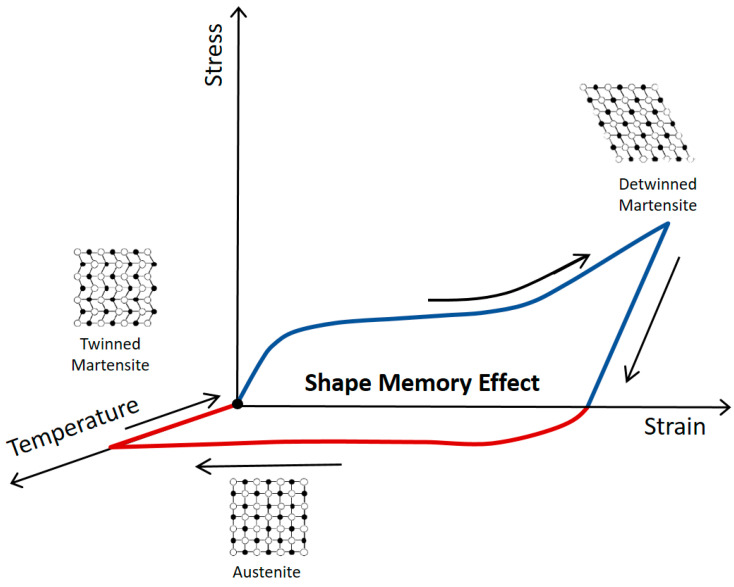
Shape memory effect of stress–strain-temperature for NiTi alloy. Temperature and stress are related to the crystal changes (Red lines; heating process, blue lines; cooling process).

The intelligent fiber utilized to fabricate the textile actuator is an SMA wire, which reacts to heat. SMA is an intelligent material with relatively high energy density. In addition, by using SMA, it is possible to lower the structure’s weight and design complexity. In order to generate macroscopic deformation, the shape memory effect (SME), a unique property of an SMA, is used in this study. The SME is a phenomenon in which a structure returns to its original shape when heated above the phase change temperature (Figure 2). The SMAs’ crystal structure is initially formed on twin martensite, and deformed martensite crystals are formed when a load is applied. When the deformed martensite is heated above the phase change temperature, the phase transformation into the austenite phase starts, and macroscopic shape recovery occurs. When SMA is cooled to a lower temperature than phase transition temperature, it is rearranged to martensite (Figure 3) [27,28,29,30,31,32,33]. The material properties of the NiTi SMA wire are presented in Table 1 [34].

### 2.3. Textile Actuator Based on Chain Structures

**Figure 4:**
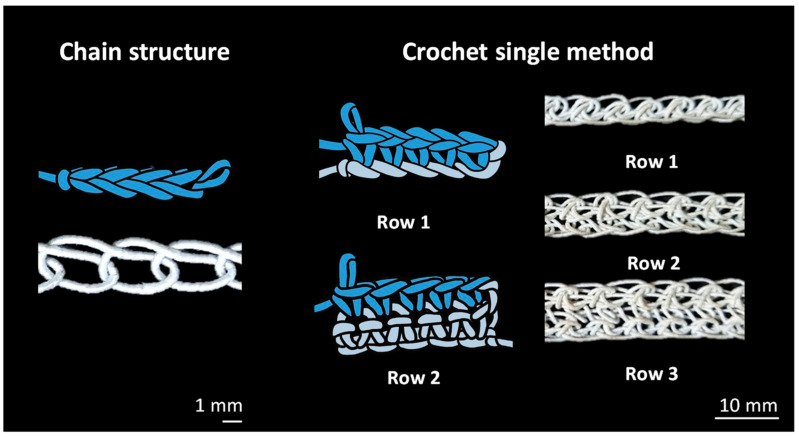
Chain actuators with different numbers of row.

Chain-structured textile actuators have varying actuation properties based on the chain size and row number. The size of the chain is varied by changing the thickness of the crochet needle used to make the chain. The following needle sizes were employed in this study: 1.75 mm, 2 mm, 2.3 mm, 2.5 mm, and 3.0 mm. Connecting the chain in a straight line creates the basic chain actuator without rows. In the case of the chain with multiple-rows, it can be produced by chains overlapping with other chain columns, and the number of rows can be added by sequentially adding columns and overlapping them. The fabricated chain actuator can be organized as shown in Figure 4. Specimen parameters for the chain actuators are shown in Table 2.

## 3. Performance Evaluations of Chain Actuators

### 3.1. Evaluations of Deformation Behaviors

**Figure 5:**
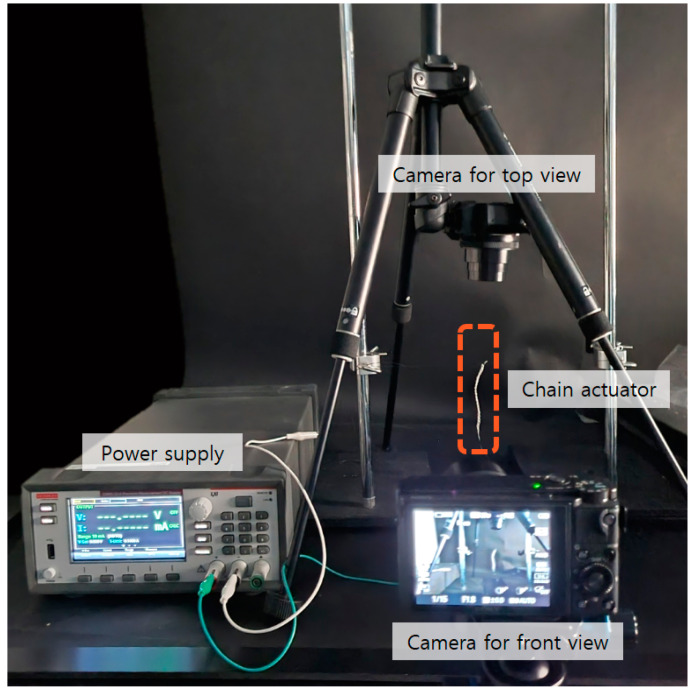
Experimental set-up for the evaluating deformation of chain actuators.

In order to study the actuating properties of the chain actuator, the deformation and actuating force were measured according to chain configuration. In this study, the Joule heating method of applying current to the SMA was used to cause the phase transition of the SMA. First, the amount of applied current was adjusted to investigate the deformation characteristics, and the associated rotation angle and vertical strain were measured. A camera was placed to record the rotation angle and vertical strain, and a power source was used to provide electricity to the actuator. Figure 5 shows the experimental setup.

**Figure 6:**
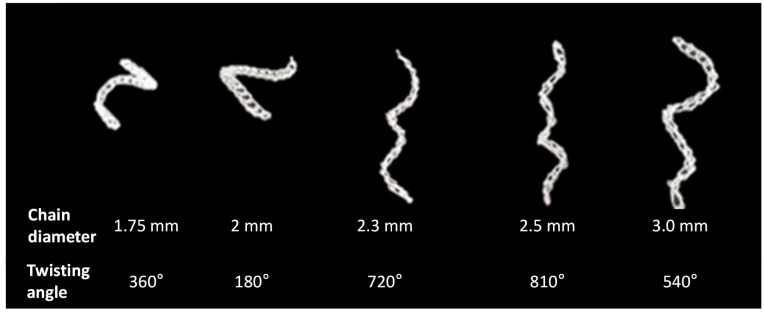
Deformation results of chain actuators according to chain diameter.

First, the torsional deformation that occurs when the chain size is different was observed, and Figure 6 shows the torsional behavior of each actuator. In the case of the actuator made with the smallest diameter crochet needle, the strength after deformation was the strongest. The actuator made using the 2.5 mm diameter crochet needle generated the most rotation. However, as the chain attains a specific size, the number of turns decreases. In this test, it was also found that a chain actuator with a needle size of 2.5 mm created more rotation than a needle size of 3.0 mm.

**Figure 7:**
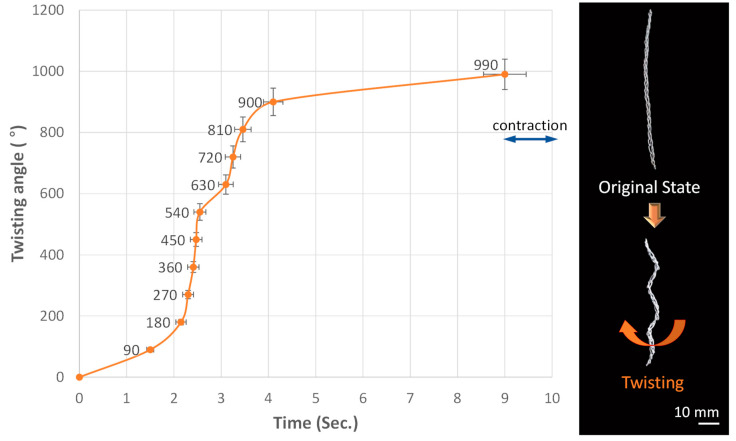
Twisting deformation of chain actuator over time.

The rotation angle and vertical strain of a chain actuator consisting of 20 chains with a needle of 2.3 mm were measured in relation to the current application time. Torsional deformation occurs due to resistance heat when a current of 0.50 A is applied to the chain actuator through the power supply. The behavior change is most significant between 2 and 4 s, and it can be confirmed that rotation behavior up to 990° is possible (Figure 7).

**Figure 8:**
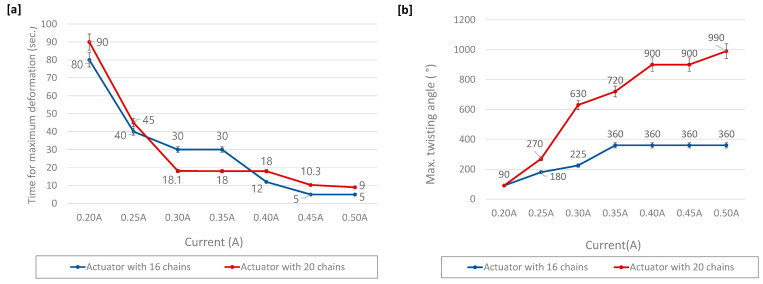
Experimental results according to the electrical current value; (**a**) time required to achieve maximum strain according to an applied current, (**b**) deformation in the twisting direction.

Next, experiments were conducted to evaluate deformations according to the conditions of the current application (Figure 8). Vertical contraction and rotation angle were determined in relation to the temperature increase rate of the chain actuator, and the temperature condition was regulated by the amount of current. The chain actuator used in the experiment has 16 chains and 20 chains, and the applied current was increased from 0.2 A to 0.5 A by 0.05 A.

To reach the maximum strain, the chain actuator consisting of 16 chains took 80 s at 0.2 A condition and 5 s at 0.5 A condition. Accordingly, it was determined that the time attaining the maximum deformation varied by about 16 times depending on the current application (Figure 8a). Upon investigating the largest achievable rotation angle, it was found that a maximum rotation angle of 360° could be obtained from the current condition of 0.35 A (Figure 8b). For the actuator with 20 chains, to reach the maximum strain, it took 90 s at 0.2 A condition and 9 s at 0.5 A condition. Comparing 0.2 A and 0.5 A, the time needed to achieve maximum strain varies by ten times and the maximum strain angle by eleven times, from 90° to 990°. In the experiment comparing the 16-chain actuator to the 20-chain actuator, the time for achieving maximum strain follows a similar decreasing trend, while the maximum twisting angle is about 2.75 times greater for the 20-chain actuator.

**Figure 9:**
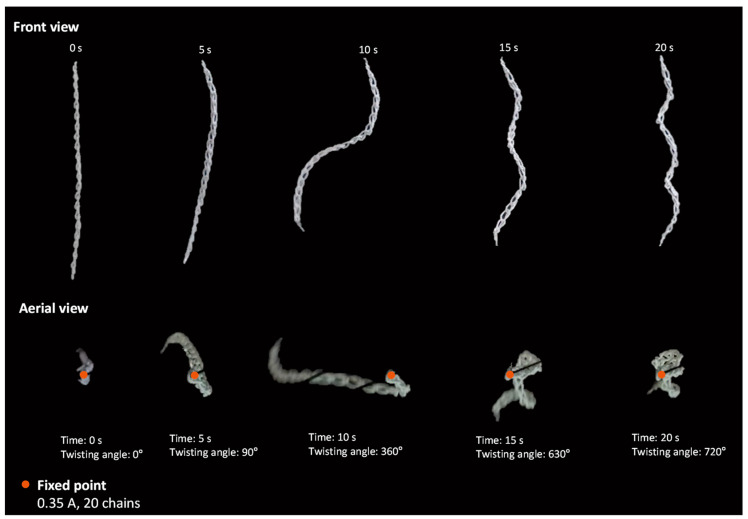
Deformation trajectories of 20 chain actuators.

Rotational deformation is prevalent in the case of the 20 chains. The majority of vertical deformation occurs simultaneously during rotation. Less than 15 percent of additional vertical deformation occurs when rotational deformation is almost completed. For 0.35 A, the greatest amount of deformation is observed between 5 and 15 s, with 90° at 5 s and 630° at 15 s (Figure 9).

### 3.2. Measurement of Actuating Forces

The actuating force due to the phase transformation generated by the electricity being applied was measured using a load cell. A thermal imaging camera was used to observe the change in response to the temperature of the actuator by application of current.

**Figure 10:**
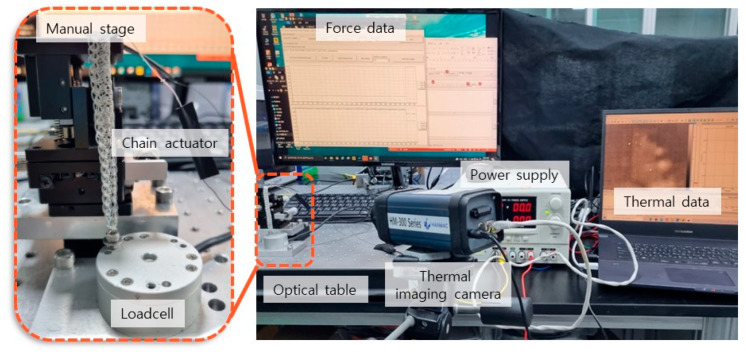
Experimental set-up for measuring force and temperature of chain actuators.

Testing was conducted with the actuator aligned perpendicular to the load cell, having been attached to the manual stage placed on the optical table. The electric current from the power supply was transmitted to the actuator, and the resultant force and temperature were monitored. Figure 10 shows the experimental setup.

**Figure 11:**
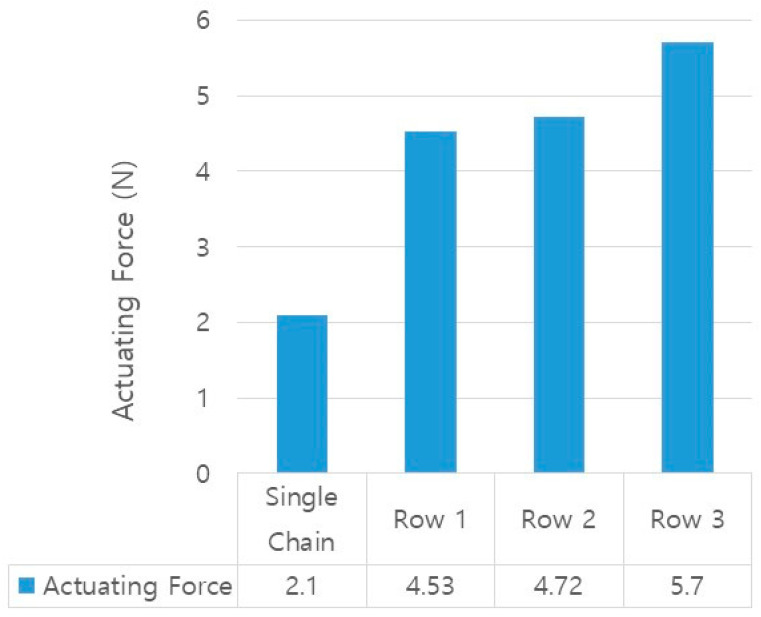
Results of actuating force with different number of rows.

Before conducting the force measurement, the actuator was fixed to the manual stage and the load cell to be in a pre-stressed condition. Considering the pre-stressed state, after calibrating the zero point of the load cell program, a phase change was induced by applying a current, and the actuating force was measured on the load cell. In this experiment, the chain actuator with different numbers of rows and twenty chains made of 2.3 mm needles was used. It is possible to connect consecutive chains in a single chain longitudinally. The longitudinal connection of the chains widens the width of the chain actuator, and the actuating force was observed as the number of rows increased. As the number of rows increased, the magnitude of the measured force increased, ranging from 2.1 N for a single chain to 5.7 N for a three-row configuration. Compared to the single chain, the specimen of row 1 showed a 2.15 times improvement in force. In addition, it was determined that the variation in width between rows 1 and 3 was approximately 1.26 times. Therefore, if the number of rows is increased and the patch is produced in a wide form along the longitudinal axis, an increase in actuating force can be expected. Figure 11 shows the results of force measurement for different rows in actuators.

**Figure 12:**
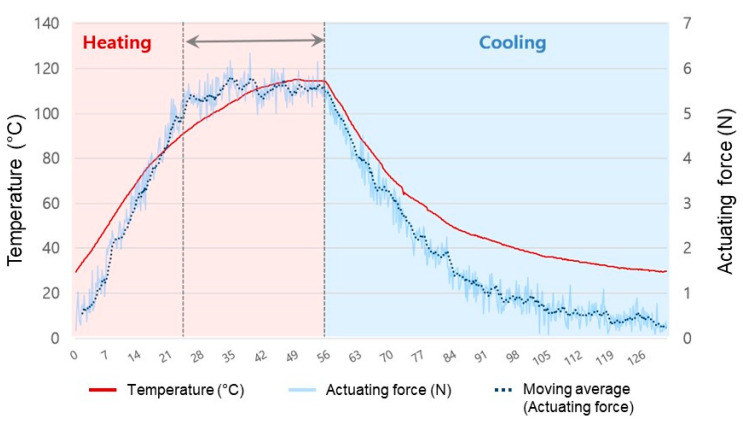
Results for measurement of actuating force and temperature over time.

To observe the detailed behaviors in chronological order, the chain actuator with three rows was tested. The temperature and generating force were recorded when 0.4 A of current was supplied (Figure 12). As the applied current increased, the actuating force and temperature of the actuator increased, and it was determined that the actuator generated an average actuating force of 5.5 N when the 0.4 A was sustained for 30 s (period from the 25-s to the 55-s in the graph). At the 25-s point, when the current reached 0.4 A, the actuator temperature was 92.8 °C and the measured actuating force was 5.7 N. The results of the experiment show that temperature and actuating force increase proportionally until the maximum actuating force is achieved. However, after attaining the maximum actuating force, the force does not change as the temperature increases. In addition, it can be seen that when the power supply is turned off, the actuator’s temperature and force decrease rapidly (period after 55 s in the graph).

**Figure 13:**
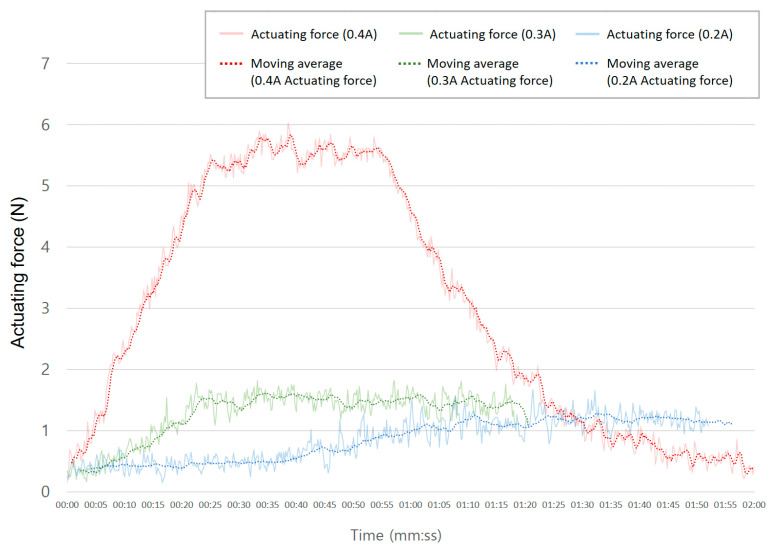
Actuating force according to the applied current.

In order to confirm the change in actuating force according to the amount of current supply, the force was measured under circumstances of current supply of 0.2 A, 0.3 A, and 0.4 A. When the maximum force was attained and convergence was identified, the supply of current was maintained for a certain amount of time and then terminated. As shown in Figure 13, the maximum actuating force at 0.2 A and 0.3 A was measured to be about 1.2 N and 1.7 N, respectively. In the case of 0.4 A, the maximum force was measured to be about 5.7 N, indicating that a current of at least 0.4 A is required to induce sufficient phase transition of the SMA.

## 4. Soft Gripper Using Textile Actuators

### 4.1. Crochet Textiles

**Figure 14:**
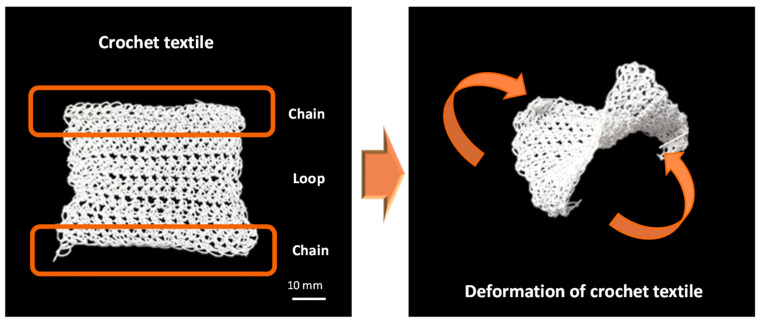
Crochet textile actuator using chain and loop patterns.

A fiber pattern comprising a chain form could be linked with a loop-type textile, and a textile manufacturing technique composed of these chains and loops is known as crochet (Figure 14). Depending on the style and number of loops linked to the chain, various textile designs may be created with crochet. Similar to the deformation of chain actuator, a twisting motion occurs when current is applied to the textile in these crochet patterns.

### 4.2. Design and Evaluation of Soft Gripper

A soft gripper was created utilizing a crochet-textile actuator that represents the torsion properties of the chain actuator. In the case of crochet-textile actuator, deformation occurs in the form of two curves as a saddle shape, and the direction of deformation follows both diagonals. The soft gripper was designed in the form of connecting four patches of the crocheted textile into one, and it was possible to pick up an object by bending in four directions.

**Figure 15:**
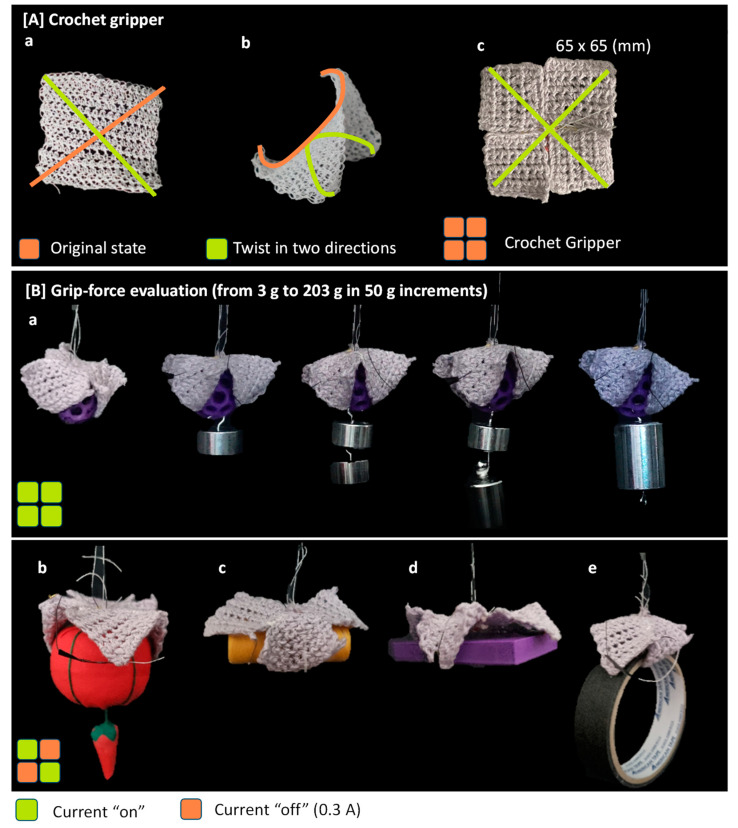
Soft grippers using textile actuators. (**A**) Crochet gripper, (**B**) Grip-force evaluation.

The lifting capability of the created soft gripper was investigated (Figure 15). The soft gripper composed of four crocheted-textile patches can change the grip mode by adjusting the current given to each patch. Upon measuring the lifting force when a current of 0.3 A was supplied, it was determined that a maximum weight of 203 g could be lifted. In addition, it was demonstrated that by operating only two patches, an item bigger than the gripper could be grasped. Furthermore, regarding grip stability, it was determined that a weight of 100 g could be gripped continuously for 120 s after the current was turned off, while a load of 5 g could be gripped for more than 72 h.

## 5. Conclusions

The behavioral properties of an intelligent textile made using SMA wire have been experimentally confirmed. Particularly, a novel design approach for textile actuators with a chain structure was developed, and the deformation and actuating force attributable to the phase transformation of the SMA was measured.

The behavioral features of chain actuators were observed by changing the size of chains and the number of chain lines connected in the transverse direction (rows). In addition, the deformation in shrinkage and twist caused by the applied current that generates the phase change was measured. As a result of the experiment, it was determined that under the Joule heating condition, torsion occurred more frequently than linear contracting motions in chain actuators.

In addition, a textile actuator was constructed using a crochet method consisting of a chain and a loop pattern, and the deformation mode was observed. Finally, a soft gripper with multiple interconnected textile patches with crochet patterns was proposed. It was possible to implement different grip modes by selectively applying current to the smart textile constituting the soft gripper, and the grasping performance was validated by targeting nearby items.

The key bullets of this study are as follows:

(a) Proposal of a smart fiber based on SMA wire and development of a soft textile actuator capable of contraction and torsional deformation by arranging it in a continuous chain configuration
(b) Experimental verification of the actuating characteristics of textile actuators by varying the chain diameter and the number of rows, which are the geometrical design conditions, and the deformation characteristics according to the magnitude of the current application that causes the phase change
(c) Fabrication of a textile soft gripper utilizing the designed chain actuator and evaluation of its grasping performance

The developed chain structure-based smart textile is a new kind of soft actuator that is capable of implementing linear contraction and twisting movements in a form composed only of fibers. In addition, it was shown that the ratio of linearity and torsion varied based on the conditions of phase transformation, indicating that different motions could be produced according to the phase control without redesigning the structure. Moreover, the proposed textile actuator is easily modifiable into a variety of forms. In particular, it can be made on a large area by combining it with a loop structure, allowing it to be used in grippers, smart suits, and other textile applications.

## Figures and Tables

**Table 1:** Material properties of SMA wires.

Parameter		Value	
Martensitic modulus	$E_{M}$	26.3	GPa
Austenitic modulus	$E_{A}$	75	GPa
Thermal coefficient	θ	0.55	GPa
Martensite start temperature	$M_{S}$	42	°C
Martensite final temperature	$M_{f}$	52	°C
Austenite start temperature	$A_{s}$	68	°C
Austenite final temperature	$A_{f}$	78	°C
Stress influence coefficient (austenite to martensite)	$C_{M}$	12	MPa/°C
Stress influence coefficient (martensite to austenite)	$C_{A}$	12	MPa/°C
Initial martensite fraction of SMA	-	1.0	-
Diameter of wire	-	200	μm

**Table 2:** Fabrication parameters for chain actuators.

Parameter	Unit	Value
Diameter of chains	mm	1.75
		2
		2.3
		2.5
		3.0
Number of rows	-	Single
		Row 1
		Row 2
		Row 3
Number of chains	-	16-chains
		20-chains

## Data Availability

Not applicable.
